# The Immune Regulation of Melanin From *Gallus gallus domesticus* Brisson Against Cyclophosphamide‐Induced Immunosuppression

**DOI:** 10.1002/fsn3.70253

**Published:** 2025-05-09

**Authors:** Jiao Liu, Haiyun Gao, Tianrui Liu, Tian Zhang, Tiegui Nan, Hongmei Li, Hiu Li, Jianliang Li, Yuan Yuan

**Affiliations:** ^1^ National Resource Center for Chinese Materia Medica China Academy of Chinese Medical Sciences Beijing China; ^2^ Jiangxi Province Key Laboratory of Sustainable Utilization of Traditional Chinese Medicine Resources Institute of Traditional Chinese Medicine Health Industry, China Academy of Chinese Medical Sciences Nanchang China; ^3^ Jiangxi Health Industry Institute of Traditional Chinese Medicine Nanchang China; ^4^ Institute of Chinese Materia Medica China Academy of Chinese Medical Sciences Beijing China; ^5^ Experimental Research Center China Academy of Chinese Medical Sciences Beijing China

**Keywords:** black‐bone silky fowl, gut microbiota, immune response, immunosuppressed mice, melanin

## Abstract

Black‐bone silky fowl (*Gallus gallus domesticus* Brisson), medicinal food homology, utilizes to enhance human immunity. However, it remains unclear whether Black‐bone silky fowl melanin (BSFM), one of its bioactive components, could affect immune function. The purpose of this study is to examine the immunoregulatory effect and the underlying mechanism of BSFM in the cyclophosphamide‐induced immunosuppressive mice model. The findings revealed that BSFM could significantly increase white blood cells (WBC) in peripheral blood; upregulate the expression of IL‐4, TNF‐α, and M‐CSF in the plasma; and reduce tissue damage. Mechanistically, proteomics has revealed that BSFM therapy substantially affected the quantity of 29 proteins (Mtatp6, Cst3, Pglyrp1, Igkc, and other targets), which mostly participate in the phosphatidylcholine catabolic process, positive regulation of type IIa hypersensitivity, lipid catabolic process, and neutrophil chemotaxis. Metabolomics indicated that BSFM reduced the levels of Octanoylglucuronide, Gly‐Gly, and N‐alpha‐acetyl‐ornithine and modulated arginine biosynthesis. Furthermore, BSFM treatment modified the composition of gut microbiota and increased the relative abundance of *Prevotella*, *S24‐7*, *Olsenella*, *Lactococcus*, *hgcl‐clade*, *Parasutterella*, and *Acetobacter*. A significant correlation modified the composition of gut microbiota among inflammation‐associated parameters, gut microbiota, and various metabolites (DMs) through Pearson correlation analysis. These findings suggest that BSFM holds promise in enhancing the human immune system and may serve as a complementary therapy in conventional chemotherapy.

## Graphical abstract

Melanin from *Gallus gallus* domesticus Brisson (BSFM) can bolster the immune system by increasing WBC in peripheral blood, stimulating the production of IL‐4, M‐CSF, and TNF‐α in the plasma, and by reducing thymus and spleen damage. BSFM therapy substantially increased the expression of immune‐related proteins, such as Cst3 and lgkc. BSFM treatment modified the composition of gut microbiota and increased the relative abundance of Prevotella, S24‐7, Olsenella, Lactococcus, hgcl‐clade, Parasutterella, and Acetobacter.

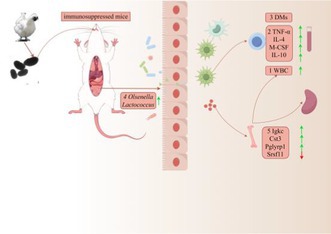

## 1 Introduction

Cy, the most widely used chemotherapeutic drug, is employed in the treatment of hematological malignancies and a range of epithelial tumors (Ahlmann and Hempel 2016). However, the high doses or long‐term use of Cy present many shortcomings and deficiencies, leading to immunosuppression (Huang et al. 2017; Viaud et al. 2013). Due to leukocytopenia, a common toxicity after chemotherapy, Cy is often used in combination with various detoxifying or protective agents to reduce its toxicity and side effects, like rhG‐CSF (Guo et al. 2014; Lichtman et al. 2001) and Vitamin B4 (Xie et al. 2017). But rhG‐CSF may cause bone pain, a major side effect, with increasing dose (Fan et al. 2020); Vitamin B4 may have a promoting effect on tumor development because of its role as a precursor of nucleic acids. Therefore, there exists a necessity to develop safer and more effective immunomodulating agents against Cy‐induced immunosuppression.

In Asian countries, chicken is a popular dietary supplement for patients suffering from cancers or other major diseases, as it had been proven to enhance immune function (Ni et al. 2022; Shih et al. 2009). Black‐bone silky fowl (*Gallus gallus domesticus* Brisson) (BSF) is one of the Chinese medicines explicitly documented in the “*Ben Cao Gang Mu* (Compendium of Materia Medica)” where it is heralded for its capacity to boost the human immune system, prevent emaciation, treat diabetes and anemia, and address women's health issues such as menoxenia and postpartum complications in China (Tu et al. 2009). Compared with other chickens, the BSF is unique due to the presence of melanin pigment in various organs, including consistent dark pigmentation in skeletal muscles (Kriangwanich et al. 2021; Muroya et al. 2000) and a wide anatomical distribution across 33 tissues and organs (Nganvongpanit et al. 2020). Previous experimental investigations have demonstrated the primary functional constituents of chicken extract as being replete with proteins, free amino acids, carnosine, and anserine (Ni et al. 2022; Xu et al. 2014). However, there remain no studies addressing the immunoregulatory benefits specifically associated with BSFM.

This study investigated the modulation effects of BSFM by assessing body weight, organ index, peripheral blood routine, and cytokines in Cy‐treated mice. Additionally, plasma metabolomics and bone marrow proteomics were carried out to elucidate complex changes in metabolites and proteins in the process of Cy‐induced immunosuppression and BSFM treatment. We also investigated whether gut microbiota interacts with the host to modulate the immune system through bacterial components and metabolites. These results may offer a theoretical foundation for the application of BSFM in treating Cy‐induced conditions in mice, thereby confirming its potential as a complementary therapy in conventional chemotherapy.

## 2 Materials and Methods

### 2.1 Materials and Reagents

**FIGURE 1:**
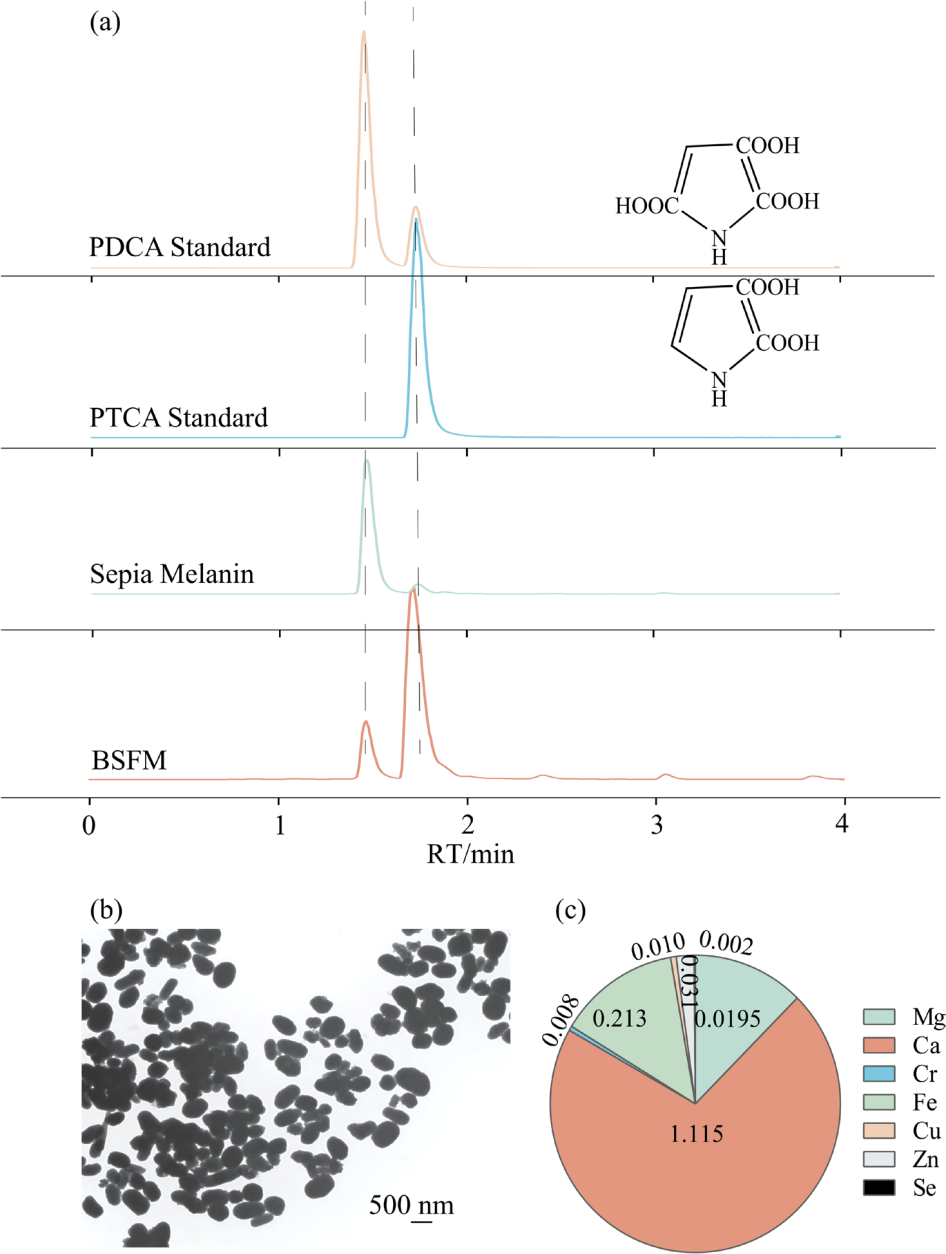
Characterization of BSFM. (a) The mass spectrum of PTCA and PDCA formed at potassium permanganate oxidation of BSFM. (b) TEM map of BSFM. (c) Kinds and content of metals in BSFM.

BSFs were purchased from Taihe Aoxin Black‐Bone Silky Fowl Development Co. Ltd. (Jiangxi, China). BSFM was extracted from BSF. Samples (100 g) were homogenized with acid (six M hydrochloric acid [HCl], 350 mL at 100°C) for 5 h using a condensation reflux device. The precipitate was obtained via centrifugation at 24,100 ***g*** for 30 min and subsequently washed three times with petroleum ether, acetic acid, and distilled water. The recovered material was dried in an oven to obtain pure BSFM. The structure of BSFM was confirmed via transmission electron microscope (TEM), liquid chromatography–mass spectrometer/mass spectrometer (LC–MS/MS), and inductively coupled plasma mass spectrometry (ICP‐MS) (Figure 1a–c and Table S1). It consists mostly of eumelanin as well as trace proteins and many metal ions, which is consistent with the reported data (Tu et al. 2009). The precipitate BSFM was preserved in a −80°C freezer for later experiments.

Cy injection was provided by Jiangsu Hengrui Pharmaceutical Co. Ltd. (Nanjing, China). rhG‐CSF was from Qilu Pharmaceutical Co. Ltd. (Jinan, China). Primary antibodies of Igkc and Cst3 were bought from UpingBio Technology Co. Ltd. (Hangzhou, China). The primary antibody of β‐actin was bought from Proteintech Group Inc. Related secondary antibodies were purchased from MediChemExpress Inc. All other chemicals were reagent grade.

### 2.2 Animals and Experimental Design

In total, 60 male ICR mice, aged 6 weeks and weighing 20 ± 2 g, were purchased from Beijing Vital River Laboratory Animal Technology Co. Ltd. (Beijing, China), under the license number SCXK[Jing] 2021‐0006. All animals were provided with sterile water and food and maintained in specific pathogen‐free conditions. All experiments followed the Guide for the Care and Use of Laboratory Animals of National Administration Regulations on Laboratory Animals of China and were approved by the Animal Ethics Committee guidelines of Institute of Chinese Materia Medica, Chinese Academy of Chinese Medical Sciences (Beijing, China, 2023B166).

**FIGURE 2:**
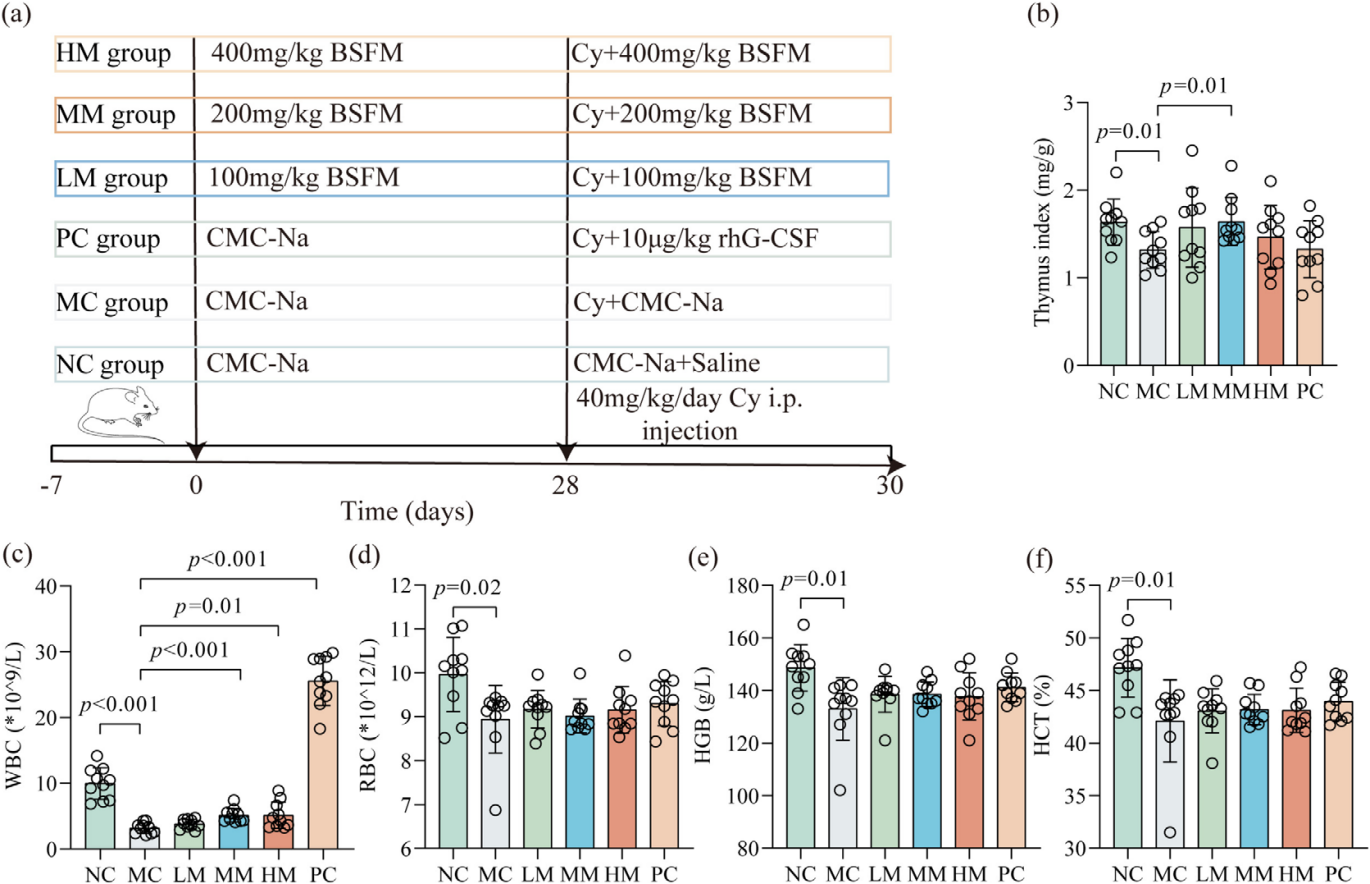
BSFM improved symptoms of Cy‐induced immunosuppression in mice. (a) Establishment of the immunosuppressive mice model and drug treatment. (b) The thymus index. (c) The number of WBC. (d) The number of RBC. (e) The content of HGB. (f) The percentage of HCT. Data were presented as the mean ± standard deviation (*n* = 10). NC, normal control; MC, Cy model; LM, low dose of BSFM (100 mg/kg); MM, medium dose of BSFM (200 mg/kg); HM, high dose of BSFM (400 mg/kg); PC, rhG‐CSF positive control.

Following 1 week of acclimatization, the mice were randomly divided into six groups (*n* = 10 in each group): the normal control group (NC), the Cy model group (MC), the Cy + low‐dose BSFM group (LM), the Cy + medium‐dose BSFM group (MM), the Cy + high‐dose BSFM group (HM), and the rhG‐CSF positive control group (PC). According to the Technical Standards for Testing and Assessment of Health Food (2023 Edition, China), the mice were administered daily oral gavage for a duration of 30 days. The NC group and MC group were treated with CMC‐Na of 0.1 mL/10 g, and the LM group, MM group, and HM group were administrated with BSFM at 100, 200, and 400 mg/kg BW, respectively, for 30 days. On the 28th, 29th, and 30th day, mice in the PC group received daily hypodermic injections of 10 μg/kg BW of rhG‐CSF. The construction of the immunosuppressive model was carried out by previous reports (Hua et al. 2017; Xu et al. 2024) and pre‐experiment. Except for the NC group, all other groups were given intraperitoneal injections of Cy at a dose of 40 mg/kg BW from day 28 to day 30 to establish the immunosuppressive model. The experiment process is shown in Figure 2a.

All animals were weighed once a week. Mice were anesthetized and sacrificed by cervical dislocation 24 h later after the last administration. Following specimens were soon taken: (1) blood: 300 μL of blood was collected from the orbital venous plexus and transferred into a 0.5 mL anticoagulation tube with EDTA for routine blood assay; (2) plasma: Plasma samples were obtained by centrifugation for 10 min (1370 ***g***, 4°C) for analyzing biochemical indicators or stored at −80°C; (3) internal organs: The livers, hearts, spleens, kidneys, and thymuses were washed in normal saline solution, dried with filter paper, and weighed immediately. Their spleens were immersed in 4% paraformaldehyde for analysis; (4) bone marrow: Their femurs were peeled off. A syringe was used to aspirate a few Hank's Balanced Salt Solution. All bone marrow was rinsed out from the femur and stored at −80°C; (5) intestinal contents: All of those were collected and stored at −80°C.

### 2.3 Blood Biochemistry and Blood Routine Examination

The whole blood was analyzed by using an auto‐hemocytometer (Sysmex XN‐1000V). The plasma IL‐4, M‐CSF, TNF‐α, and IL‐10 were analyzed and determined using a Mouse Premixed Multi‐Analyte Kit (R&D Systems).

### 2.4 Organ Coefficient

Organ coefficient was calculated by the following equation: organ coefficient = [organ wet weight (g)/body weight (g)] × 100% (Liang et al. 2021).

### 2.5 Histological Observation

Tissue specimens of the spleen were prepared according to a previously described method (Wu et al. 2023). In brief, the spleen tissues were fixed using 4% paraformaldehyde for at least 24 h. After fixation, the tissues were embedded in paraffin wax and sliced into 4 μm sections using a Leica microtome (LEICA RM2016). To evaluate the splenic histopathological changes, hematoxylin–eosin (H&E) staining was conducted and subsequently examined under a microscope (NICON Eclipse Ci). In addition to the H&E‐stained sections, other paraformaldehyde‐fixed spleen tissues were incubated with primary antibody CD3 (Proteintech) at 37°C for 2 h, then washed with PBS 3 times, and finally used for immunohistochemical analysis. Image J software (version 1.47) was used to analyze each image to obtain the area of white pulp and the CD3^+^‐positive area.

### 2.6 Plasma Metabolomics

Mix plasma (50 μL) with 200 μL of extract solution (methanol, containing isotopically labeled internal standard mixture) and vortex for 2 min. The samples were allowed to stand for 10 min and then centrifuged for 15 min (14,000 ***g***, 4°C). Finally, transfer the obtained supernatant for LC–MS/MS analysis.

LC–MS/MS analysis was conducted using an Ultimate 3000 HPLC system (Thermo Scientific) and Q Exactive mass spectrometer (Thermo Scientific). Chromatographic separation was performed on a Waters ACQUITY UPLC BEH C18 column (2.1 mm × 100 mm, 1.7 μm) and an HSS T3 column (2.1 mm × 100 mm, 1.8 μm), with a flow rate of 0.35 mL/min^−1^. The column oven was set to 50°C. The mobile phase comprised 0.1% formic acid in water (A) and 0.1% formic acid in acetonitrile (B). The gradient program employed was as follows: 0–1 min, 5% B; 1.1–11 min, 5%–100% B; 11.1–13 min, 100% B; 13.1–15 min, 5% B. The injection volume was set at 5 μL.

The mass spectrometer was operated under the following conditions: the sheath gas flow rate at 35 Arb, the aux gas flow rate at 8 Arb, capillary temperature set to 320°C, with a full MS resolution of 70 000, and an MS/MS resolution of 17 500. The collision energy was adjusted to 20 and 40 in NCE mode, and the spray voltage was set to either +3.8 kV for positive ion mode or −3.0 kV for negative ion mode.

The model was developed using principal component analysis (PCA). Metabolites with *p*‐value < 0.05 and variable importance in projection (VIP) values > 1 were considered statistically significant.

### 2.7 DIA‐Based Quantitative Proteomics

In this study, protein quantification and DIA techniques were employed to analyze proteomics. First, the myeloid tissues of nine mice were randomly chosen from each of the NC, MC, and HM groups. Following protein extraction, quality assessment, and enzymatic digestion, the peptide segments were separated via HPLC. The eluted peptide was redissolved in mobile phase A (consisting of water and 0.1% formic acid), subjected to centrifugation at 14,000 ***g*** for 20 min, and subsequently injected for analysis using LC–MS/MS.

### 2.8 16S rRNA Sequencing of Gut Microbiota

Total genomic DNA was extracted from fecal samples of mice using the Omega mag‐bind soil DNA Kit (Omega). DNA quality was monitored on 1% agarose gels. The detailed methods have been described previously (Liu et al. 2016). The amplification used primers 338F (5′‐ACTCCTACGGGAGGCAGCAG‐3′) and 806R (5′‐GGACTACHVGGGTWTCTAAT‐3′). The concentration and purity of DNA were monitored on 2% agarose gels. Amplicon libraries were created employing the TruSeq Nano DNA LT Library Preparation Kit (Illumina) and subsequently sequenced on an Illumina Miseq platform.

The operational taxonomic units (OTUs) were grouped into clusters with a 97% identity using an open reference approach (USEARCH 8.1.1861), and taxonomic identities were assigned to individual OTUs using the SILVA SSU r138 RefNR database. The RDP classifier was used for aligned sequences. Alpha diversity was measured by calculating the diversity indexes of Chao1, ACE, Shannon, and Simpson. The relative abundances at the phylum and genus levels were also analyzed.

### 2.9 Western Blotting Measurement

Total protein of myeloid tissues was extracted and quantitated using RIPA lysis buffer and BCA protein assay kit (Beyotime), respectively. Following concentration adjustment with deionized water and 6 × protein loading buffer (containing DTT), the samples were denaturized at 95°C for 10 min. The denatured samples (10 μg) were separated by 4%–20% SDS‐PAGE and transferred onto PVDF membranes. The membrane was blocked and incubated with primary antibodies (1: 1000 dilution) at 4°C overnight. The corresponding primary antibodies were applied: anti‐Igkc, anti‐Cst3, and anti‐β‐actin. The membrane was washed with TBST for 3 times and incubated with secondary antibodies at RT for 1 h. Protein signals were visualized by ultra high sensitivity ECL kit (MedChemExpress) and Bio‐Rad Gel Imaging Systems (Bio‐Rad).

### 2.10 Statistical Analyses

All data were expressed as the mean ± SEM. Use one‐way ANOVA and *LSD* test to analyze intergroup differences. Spearman correlation was conducted to analyze the relationships between biochemical indicators (BIs), genera, and differential metabolites (DMs) in the MC and HM groups. The critical *p*‐value was set at *p* < 0.05. Statistical analyses were performed via GraphPad Prism V.8.0.2.

## 3 Results

### 3.1 Sample Composition Analysis

As clearly shown in Figure 1b, under a magnification of 15,000×, numerous elliptical polymer particles and attached proteins were visible, characterized by their irregular, elliptical edges. After the observation at the nanoscale, the chemical analyses of the components of BSFM were performed using ICP‐MS and LC–MS/MS (Figure 1a,c). The compositional analysis revealed that BSFM consisted of copper, zinc, iron, magnesium, cadmium, calcium, and selenium. The calcium content is the highest among them (Figure 1c and Table S1). BSFM is mainly composed of 5,6‐dihydroxyindole (DHI) and 5,6‐dihydroxyindole‐2‐carboxylic acid (DHICA) units with different states. The isolated pigment contained eumelanin, trace proteins, and various metal ions, with a high content of calcium, magnesium, iron, and zinc, which is consistent with the reported data (Tu et al. 2009).

### 3.2 BSFM Treatment Improves the Peripheral Hemogram and Thymus Index

Previous studies have shown that high‐dose Cy can reduce the number of WBC in blood (Chu et al. 2020). So after 3 days of Cy intervention, the peripheral blood was determined. Notably, the levels of WBCs, RBCs, HGB, and HCTs in the HM group were significantly higher than those in the MC group (*p* < 0.05) (Figure 2c–f). A comparison with the MC group shows that WBC levels in HM groups were also increased (*p* < 0.05).

Thymus and spleen are important organs that play a significant role in the regulation of immune responses. Degeneration and atrophy of these organs could harm the function of the entire immune system (Niu et al. 2020; Wen et al. 2020). Compared with the MC group shows that other organ indexes did not significantly differ (Table S2), whereas the thymus index of both the NC and MM groups exhibited a significant increase (*p* < 0.05) (Figure 2b).

### 3.3 BSFM Treatment Adjusts Cytokine Balance and Promotes Splenic Lymphocyte Proliferation

**FIGURE 3:**
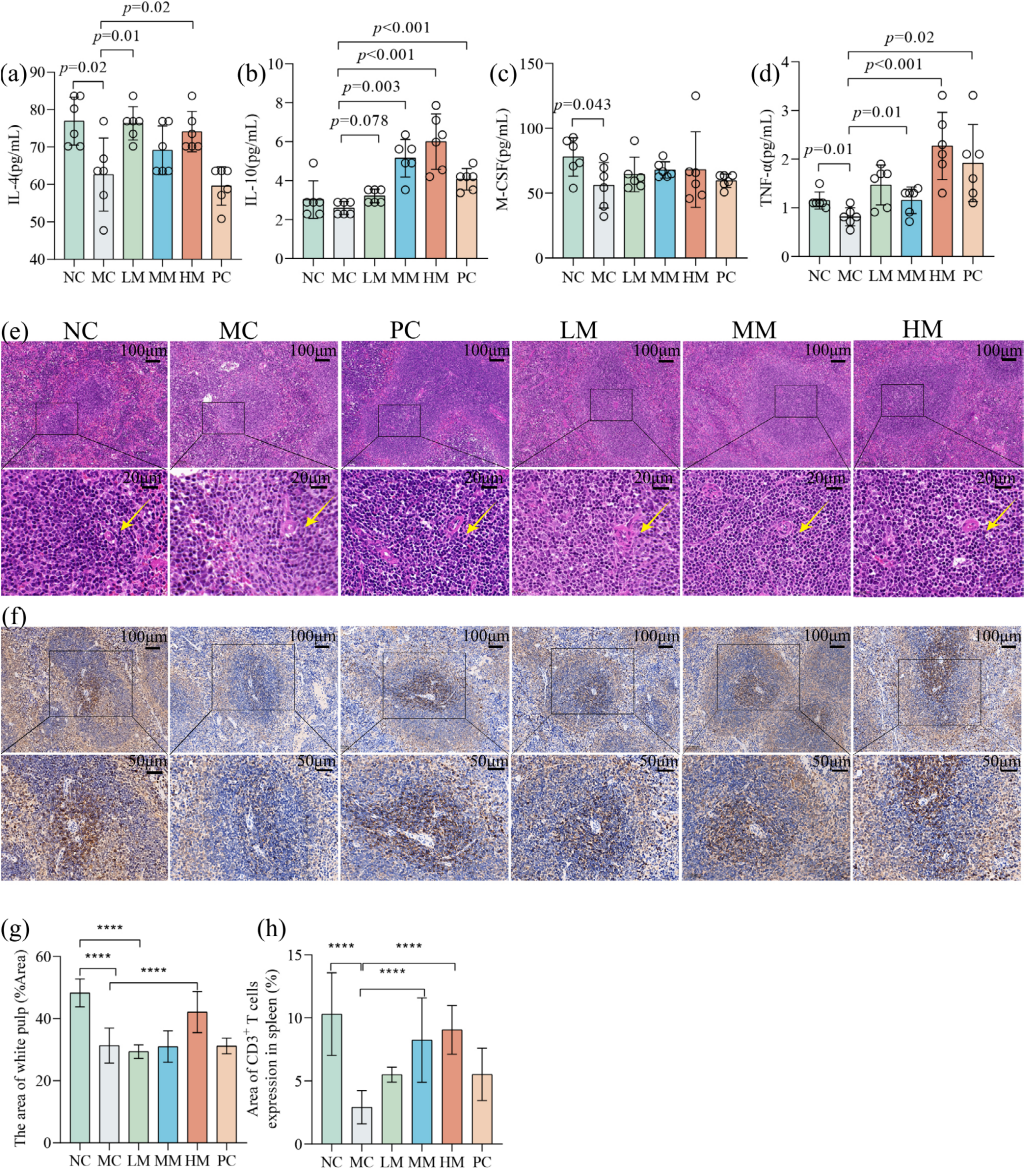
BSFM treatment adjusts cytokines balance and promotes splenic lymphocyte proliferation. (a) The levels of IL‐4. (b) The levels of IL‐10. (c) The levels of M‐CSF. (d) The levels of TNF‐α. (e) H&E‐stained images of spleen tissues (scale bars: 100 μm, 20 μm); yellow arrow in figure was marked as the location of spleen cell. (f) Representative immunohistochemistry profiles of CD3^+^ T in spleen with a scale bar of 100 μm and 50 μm. (g) Quantitation of the area of white pulp per field using Image J software. (h) Quantitation of positive area per field using Image J software. Data were presented as mean ± standard deviations (*n* = 6–10). NC, normal control; MC, Cy model; LM, low‐dose of BSFM (100 mg/kg); MM, medium‐dose of BSFM (200 mg/kg); HM, high‐dose of BSFM (400 mg/kg); PC, rhG‐CSF positive control. ****p < 0.001.

Cytokines are responsible for promoting and regulating immune response (Ferreira et al. 2018). Compared with the MC group, the results show that IL‐4, TNF‐α, M‐CSF, and IL‐10 levels in HM groups were increased (*p* < 0.05). In the PC group, the administration of rhG‐CSF also restored the levels of all cytokines except M‐CSF (*p* < 0.05) (Figure 3a–d).

The spleen serves as a filter for blood and an essential organ for monitoring the circulation of lymphocytes. The circulation of systemic blood via the white pulp and marginal zone facilitates an effective acquired immune response. The strategic positioning of leukocytes in the marginal zone ensures nonspecific immunity (Aliyu et al. 2021). To further corroborate the impact of BSFM on immunosuppression, we stained the spleen with H&E and collected spleens for immunohistochemical detection of CD3^+^ T cells. In the NC group, spleen cells were tightly and orderly arranged. Conversely, in the MC group, the spleen cells appeared sparse and disorganized. The spleen in the HM exhibited a morphology similar to that of the NC group, with closely arranged lymphocytes (Figure 3e). The high‐dose BSFM can increase the white pulp area of the spleen in comparison with the MC group (Figure 3g). BSFM substantially raised the CD3^+^ T cells in the spleen compared to the MC group (Figure 3f,h), indicating that BSFM could promote the recovery of spleen immune function.

### 3.4 BSFM Treatment Regulates Protein Expression in Bone Marrow

**FIGURE 4:**
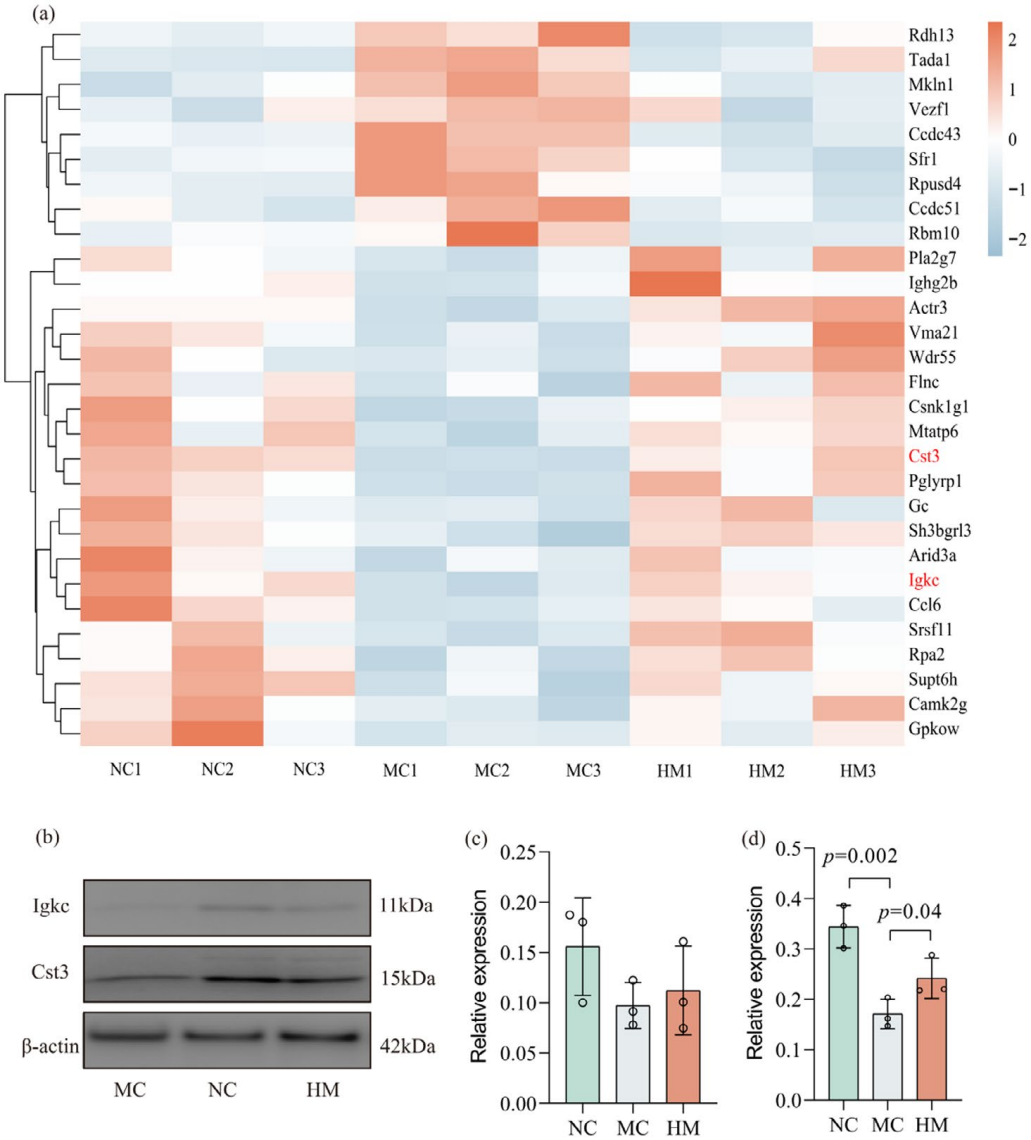
BSFM regulated the development of myeloid tissue of Cy‐induced immunosuppressed mice. (a) Heatmap of the common DEPs. (b) The expression of Igkc and Cst3 in myeloid tissue using Western blot. *n* = 3 in each group. (c) The relative levels of Igkc. (d) The relative levels of Cst3. NC, normal control; MC, Cy model; HM, high‐dose of BSFM (400 mg/kg).

WBCs can combat external microorganisms, but they have a short lifespan and rely on bone marrow stem cells to continuously differentiate complement (Han et al. 2019). Bone marrow, as a highly active hemopoietic tissue, is characterized by its rapid cell division and its extreme sensitivity to radiation and chemotherapeutic agents, which can lead to great damage (Liu et al. 2014). Hence, the protein expression profile of myeloid tissue from mice in the NC, MC, and HM was examined using LC–MS/MS with the DIA data acquisition method. A total of 4839 trusted proteins were identified, of which 4740 were quantified. Anatomizing differentially expressed proteins between NC vs. MC and MC vs. HM, only 29 proteins showed a retracement trend, with 20 upregulated and nine downregulated (Figure 4a and Table S3). Results from Gene Ontology (GO) and Kyoto Encyclopedia of Genes and Genomes (KEGG) pathway are presented in Figure S2. Pathways of neurodegeneration‐multiple diseases and amyotrophic lateral sclerosis were identified as the most significantly impacted KEGG pathways in developing myeloid tissue of Cy‐induced immunosuppressed mice. Also, the phosphatidylcholine catabolic process, positive regulation of type IIa hypersensitivity, lipid catabolic process, and neutrophil chemotaxis were the most affected biological processes. Cst3 is widely expressed in various body fluids and tissues (Cai et al. 2022; Mohd Tahir et al. 2020), and it is important to modulate immune cell activation (Shu et al. 2018). Igkc is the immunoglobulin kappa chain (Schmidt et al. 2012). The Western blot of the expression of Igkc and Cst3 displayed a similar tendency (Figure 4b–d).

### 3.5 BSFM Treatment Regulates Cy‐Induced Abnormal Metabolic Profiling

**FIGURE 5:**
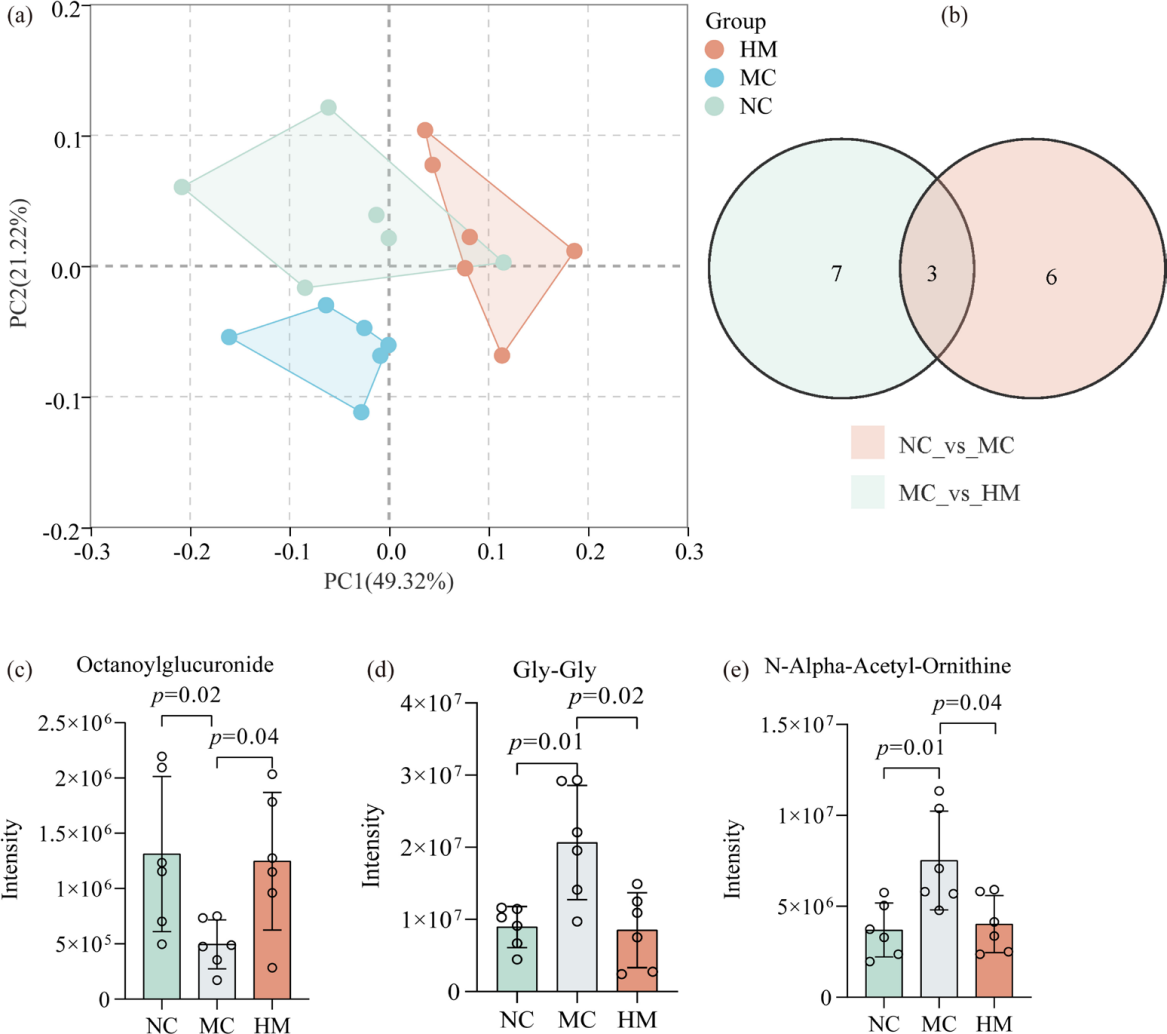
BSFM regulated plasma metabolism (*n* = 6). (a) Principal component analysis (PCA) results of NC, MC, and HM groups. (b) Venn diagram showing the overlap of the metabolites identified in the plasma among the three groups. (c–e) Differential metabolites characterized in the plasma and their change trends after BSFM treatment. NC, normal control; MC, Cy model; HM, high dose of BSFM (400 mg/kg).

In this study, untargeted metabolomics was performed to analyze the metabolic profiles of the plasma and assess whether the observed effect of BSFM was related to changes in metabolites. PCA plot illustrated clear separations among NC vs. MC vs. HM (Figure 5a). Based on the results of PCA, the metabolites with fold change ≥ 1.5 or fold change ≤ 0.5 and VIP ≥ 1 were identified as differential metabolites. Nine differential metabolites were identified between the NC and MC groups, including 1‐[(2R,3S,5R)‐3,4‐Dihydroxy‐5‐(hydroxymethyl)oxolan‐2‐yl]pyrimidine‐2,4‐dione, 3‐Hydroxycapric acid, N‐Alpha‐Acetyl‐Ornithine, L‐Threonine, 6‐Phosphogluconic acid, Mono‐ethylhexylphthalate, Octanoylglucuronide, 2‐AMINOISOBUTYRATE, and Gly–Gly. Ten DMs were observed in the HM treatment group compared to the MC group, including one upregulated and nine downregulated metabolites, mainly involving Octanoylglucuronide, Indole‐3‐aldehyde, 2‐methyladipic acid, GLUCURONATE, D‐Gluconic acid sodium salt, D‐Mannose‐6‐phosphate barium salt hydrate, Homovanillic acid sulfate, Gly–Gly, N‐alpha‐acetyl‐ornithine, and Melatonin. Its detailed MS/MS information and intensity data are shown in Table S4. We comprehensively analyzed these metabolites and assumed that three metabolites may be the key biomarkers for BSFM in treating Cy (Figure 5b–e).

### 3.6 BSFM Treatment Reversed Cy‐Induced Gut Dysbiosis

**FIGURE 6:**
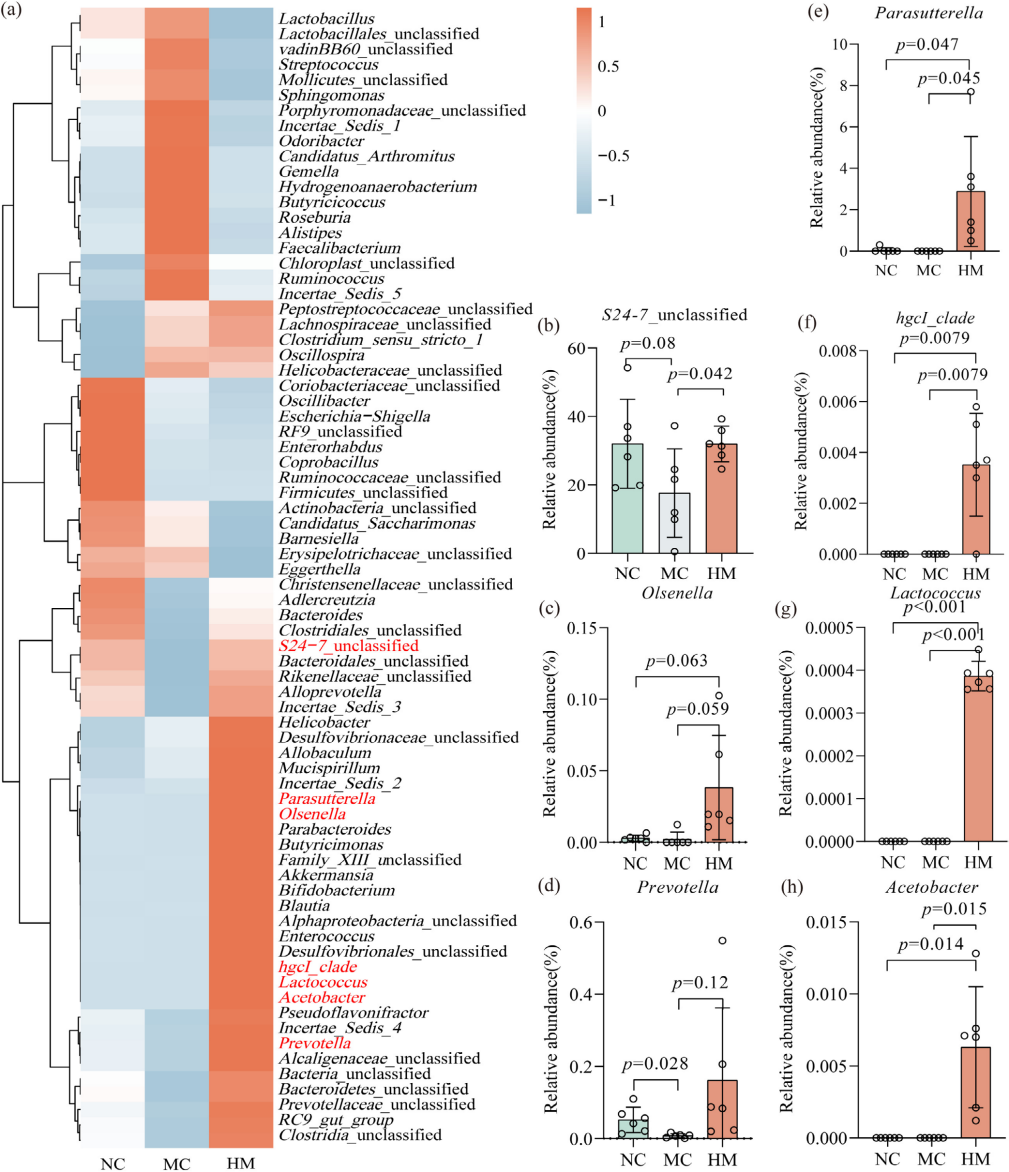
Effects of oral administration of BSFM on gut microbiota composition. (a) Genus level. (b–h) Seven species of bacteria at the genus level. NC, normal control; MC, Cy model; HM, high dose of BSFM (400 mg/kg).

The gut microbiota plays a critical role in upholding intestinal homeostasis, and it has been observed that Cy can alter gut microbiota composition (Viaud et al. 2013). At the phylum level, the microbial community was mainly dominated by *Firmicutes*, *Bacteroidetes*, *Proteobacteria*, and *Actinobacteria* (Figure S4). At the genus level (Figure 6a), mice in the MC group showed significantly lower abundances of *Prevotella* compared to the NC group. In the HM groups, the abundance of *Olsenella*, *Prevotella*, *S24‐7*, *Lactococcus*, *hgcl_clade*, *Parasutterella*, and *Acetobacter* was much higher than that in the NC and MC groups (Figure 6b–h and Table S5).

### 3.7 Correlation Analysis

**FIGURE 7:**
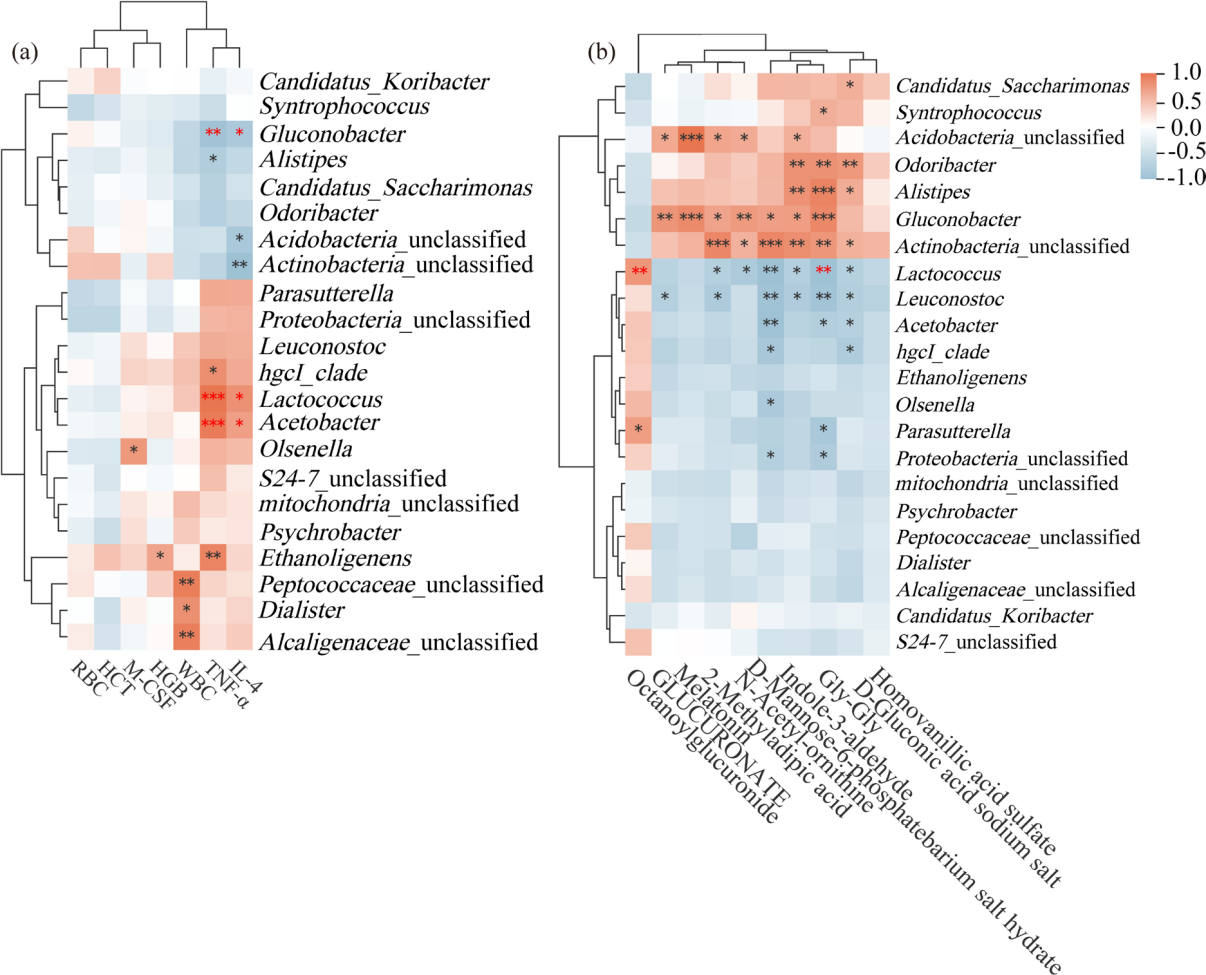
Statistical analyses using the Spearman correlation coefficient were performed to examine the relationship. (a) BIs and DMs. (b) DMs and genus. The intensity of color represents the degree of association. *p < 0.05, **p < 0.01, ***p < 0.001.

Spearman correlation analysis was conducted on BIs, genera, and DMs (Figure 7). TNF‐α and IL‐4 presented positive correlations with the relative abundances of *Lactococcus* and *Acetobacter*. There were negative correlations with the relative abundances of *Gluconobacter*. The relative abundances of *Lactococcus* displayed positive correlations with Octanoylglucuronide and negative correlations with Gly‐Gly and N‐alpha‐acetyl‐ornithine.

## 4 Discussion

Cy, a nonspecific cell cycle alkylating agent, primarily targets the S‐phase, exerting its cytotoxic influence by disrupting DNA synthesis (Priyadarshini 2021). It can deplete hematopoietic stem cells within the marrow and circulating peripheral blood cells, thereby lending to hematopoietic suppression and immune deficiency (Tiev et al. 2014). In Cy‐induced immunosuppressed mice, the levels of white blood cells and the expression of IL‐1β, IL‐4, IL‐6, as well as TNF‐α in serum were reduced (Qi et al. 2019). Additionally, Cy treatment significantly diminishes spleen and thymus indices, inducing apoptosis in these vital immune organs (Tang et al. 2022; Zhang et al. 2022). Recent findings suggest that Cy can disrupt the mucus layer of the gut, altering the gut microbiota and resulting in the accumulation of monocytes in the lamina propria, as well as the translocation of Gram‐positive bacteria to mesenteric‐positive lymph nodes and spleen (Viaud et al. 2013).

As the utilization of Cy continues to rise in China (https://www.menet.com.cn/), the search for effective immunomodulating agents remains imperative. Traditional Chinese Medicine and functional foods emerge as promising alternatives for immune modulation. For instance, the combination of Korean Red ginseng and *Colla corii asini* can improve the myelosuppression of Cy and enhance the hematopoietic effect (Lee et al. 2021). Similarly, glucan from the fruit body of *Dictyophora rubrovolvata* ameliorated Cy‐induced immunosuppression via inducing CD4^+^ T cell proliferation and differentiation (Wu et al. 2023). The selenium‐enriched soybean protein (Zhang et al. 2021) and ovotransferrin (Zhu et al. 2019) could also effectively boost the immune ability of immunosuppressed mice. BSF has been used for a long time in Traditional Chinese Medicine to enhance human immunity. Components such as chicken essence (Ni et al. 2022) and carnosine (Xu et al. 2014), derived from chicken, have been utilized in the treatment of patients suffering from immunosuppression. However, the immunoregulatory effects and mechanism of action of BSFM, a functional ingredient derived from the BSF, remain largely unexplored. Recent experimental evidence supports that melanin derived from *Lachnum singerianum* can bolster the immune system by stimulating the production of IL‐2 and TNF‐α in the plasma and by increasing thymus and spleen indexes (Ye et al. 2019).

By analyzing bone marrow proteins, Cst3 and Igkc attracted our attention. A nonglycosylated cysteine protease inhibitor prevents bone resorption (Strålberg et al. 2013), modulates inflammation‐driven cell death, and counteracts the activity of cathepsins (Chu et al. 2020). Igkc serves as a representative marker of the B‐cell gene signature and is linked to improved prognosis in breast cancer and multiple myeloma (Tangen et al. 2015; Whiteside and Ferrone 2012). Western blot validation confirmed the expression of Igkc and Cst3 in myeloid tissues. These findings demonstrate that BSFM restored immune responses in plasma and spleen and enhanced the expression of immunoregulatory proteins in bone marrow to counteract Cy‐induced immunosuppression.

Given BSFM's high molecular weight and insolubility in common solvents, its direct entry into the bloodstream is limited, leading us to hypothesize its effects may be mediated via gut microbiota alterations. We employed 16S rRNA amplicon sequencing to assess changes in the gut microbial diversity, using indices like Shannon, Chao1, ACE, and Simpson. Our data indicated increased microbial diversity in the BSFM‐treated group (Figure S3). Taxonomic analysis revealed an enrichment of bacteria such as *Prevotella*, *S24‐7*, *Olsenella*, *Lactococcus*, *hgcl‐clade*, *Parasutterella*, and *Acetobacter*, following treatment. These bacterial groups are implicated in various beneficial metabolic and immune functions. *Prevotella*, a diverse genus of gram‐negative anaerobic bacteria, plays a role in glucose homeostasis and host metabolism (Asnicar et al. 2021; Tett et al. 2021). The *S24‐7* family, which is prevalent in the gut microbiota of mice, was found to be positively associated with a delayed onset of diabetes and negatively correlated with SBP, LPE 18:0, LPE 18:1, and LPE 18:2 in cases of hypertension (Krych et al. 2015; Toral et al. 2019; Zhu et al. 2023). *Olsenella* species have been shown to enhance the efficacy of anti‐CTLA‐4 treatment and boost the activation of CD4^+^ and CD8^+^ T cells in four mouse models of cancer (Mager et al. 2020). *Lactococcus* was beneficial for neuroprotection in HFD‐induced neuroinflammation (Yuan et al. 2019). To further confirm the impact of microbiota on metabolic products, we undertook a metabolomic analysis.

Metabolomic analysis identified three key metabolites—Octanoylglucuronide, Gly‐Gly, and N‐alpha‐acetyl‐ornithine—associated with BSFM treatment. Octanoylglucuronide, a medium‐chain fatty acid conjugate of glucuronate, has been detected in the urines of pediatric patients with inflammatory bowel disease as well as in individuals with congenital metabolic disorders (Duran et al. 1985; Yamamoto et al. 2021). Dipeptide Gly‐Gly is the well‐known γ‐glutamyl acceptor and exhibits anticonvulsant activity (Samuels et al. 1983). N‐alpha‐acetyl‐ornithine is among the metabolites of amino acids and can be processed by distinct differential ontology genes within the intestinal metagenome (Devi et al. 2019; Wu et al. 2022). These results showed that BSFM could regulate metabolites in Cy‐induced immunosuppressive mice via various mechanisms including pentose and glucuronate interconversions and arginine biosynthesis. Relative studies have proven a positive correlation between *Parasutterella* abundance and treatment of ulcerative colitis (Yang et al. 2023). Despite the absence of significant changes in gut microbiota α‐diversity, Spearman's correlations linked *Lactococcus* with plasma cytokines and DMs, indicating potential metabolic impacts. Nevertheless, more detailed investigations are necessary to elucidate the precise mechanisms of BSFM on gut microbiota.

## 5 Conclusions

This study elucidates the protective effects of BSFM on Cy‐induced immunosuppressive mice models. The study highlights BSFM's capacity to counteract immunosuppressive conditions, primarily through enhancing peripheral blood cell counts, modulating cytokine secretion, and fostering the proliferation of splenic lymphocytes. Additionally, BSFM also plays a crucial role in regulating bone marrow signaling proteins and optimizing plasma metabolism, while also promoting a balanced and enriched gut microbiota composition. These findings provide a promising basis for the use of melanin derived from black‐bone silkie chicken as an adjunctive therapy to alleviate the side effects associated with Cy‐induced immunosuppression and pave the way for its possible development as a functional nutritional supplement.

## Author Contributions

**Jiao Liu:** conceptualization (equal), methodology (equal), writing – original draft (equal). **Haiyun Gao:** data curation (equal). **Tianrui Liu:** formal analysis (equal), methodology (equal). **Tian Zhang:** data curation (equal). **Tiegui Nan:** data curation (equal). **Hongmei Li:** methodology (equal), supervision (equal). **Hiu Li:** funding acquisition (equal), supervision (equal). **Jianliang Li:** supervision (equal), writing – review and editing (equal). **Yuan Yuan:** funding acquisition (equal), supervision (equal).

## Conflicts of Interest

The authors declare no conflicts of interest.

## Supporting information


**Table S1.** Determination of mineral element content (X ± S, *n* = 3).
**Table S2.** Organ index.
**Table S3.** Expression of target proteins after application of BSFM treatment.
**Table S4.** Differential metabolites characterized in plasma and their change trends after melanin treatment.
**Table S5.** The abundance values of bacteria at the genus level.
**Figure S1.** Changes in body weight of mice.
**Figure S2.** Function analyses of common DEPs between NC vs. MC and MC vs. HM.
**Figure S3.** Alpha diversity indices.
**Figure S4.** Phylum level.

## Data Availability

The metabolomics, DIA proteomics, and 16S rRNA sequencing data have been deposited at CNCB, bigd.big.ac.cn/bioproject/ (accession no. PRJCA034074).
