# Supplementary material for: The Immune Regulation of Melanin From Gallus gallus domesticus Brisson Against Cyclophosphamide‐Induced Immunosuppression
Source: Food Sci Nutr. 2025 May 9;13(5):e70253. doi: 10.1002/fsn3.70253 (PMC12064410; doi:10.1002/fsn3.70253)
Supplement: Supplementary file 1 — Table S1. Determination of mineral element content (X ± S, n = 3). Table S2. Organ index. Table S3. Expression of target proteins after application of BSFM treatment. Table S4. Differential metabolites characterized in plasma and their change trends after melanin treatment. Table S5. The abundance values of bacteria at the genus level. Figure S1. Changes in body weight of mice. Figure S2. Function analyses of common DEPs between NC vs. MC and MC vs. HM. Figure S3. Alpha diversity indices. Figure S4. Phylum level. [file FSN3-13-e70253-s001.docx]

**The immune regulaton effects of melanin from *Gallus gallus domesticus* Brisson against cyclophosphamide-induced immunosuppression**

Jiao Liu^1, 2^, Haiyun Gao^2, 3^, Tianrui Liu^2, 3^, Tian Zhang^1^, Tiegui Nan^1^, Hongmei Li^4^, Hiu Li^2, 3, 4^, Jianliang Li^1^, Yuan Yuan^5^^[[1]](#footnote-0)^

1 National Resource Center for Chinese Materia Medica, China Academy of Chinese Medical Sciences, Beijing 100700, China

2 Jiangxi Province Key Laboratory of Sustainable Utilization of Traditional Chinese Medicine Resources, Institute of Traditional Chinese Medicine Health Industry, China Academy of Chinese Medical Sciences，Nanchang 330115, China

3 Jiangxi Health Industry Institute of Traditional Chinese Medicine, Nanchang 330115, China

4 Institute of Chinese Materia Medica, China Academy of Chinese Medical Sciences, Beijing 10

0700, China

5 Experimental Research Center, China Academy of Chinese Medical Sciences, Beijing 100700, China

**Supplementary Materials and Methods**

**Preparation of BSFM.**

**S. 1 TEM**

Take a small amount of dried, refined melanin with an appropriate volume of ethanol. Use ultrasound to uniformly disperse the sample within mixture, creating a homogenous solution. Utilize a glass capillary to extract 2-3 droplets of the mixed solution and transfer them onto a microgrid. Transmission electron microscope (TEM) photos were botained from H-7650 (Hitachi) with an operating voltage of 75 KV.

**S. 2 LC-MS/MS**

The oxidation products of BSFM were extracted and analyzed using LC-MS/MS, following the previously described protoco (Sun et al., 2008). Briefly, precisely measured 10 mg of crude melanin, combine it with 8.6 mL of 1 moL/L K_2_CO_3_ and 0.8 mL of 30% H_2_O_2_, and then immerse the mixture in a 100 ℃ water bath for 20 minutes. Subsequently, cool the mixture under running water and halt the reaction by adding 0.4 mL of 10% Na_2_SO_3_. Adjust the pH to approximately 1.0 using 6 mol/L HCl. Following centrifugation at 9500 r/min, collect the supernatant, evaporate it to dryness using nitrogen gas, redissolve it in the mobile phase, filter it through a 0.45 μm organic membrane, and proceed with LC-MS/MS analysis.

LC-MS/MS analyses were performed on an Aquity UPLC system (Waters Corporation) coupled with a Q Exactive mass spectrometer (Thermo Scientific). The chromatographic separation was performed using a Waters ACQUITY UPLC BEH C18 column (2.1 mm × 100 mm, 1.7 μm), with a flow rate of 0.4 mL min^-1^. The column oven was maintained at a temperature of 40 ℃. The mobile phase was composed of 0.1% formic acid in water (A) and 0.1% formic acid in methanol (B). The gradient program was excuted as follows: 0-3 min, 5%-25% B; 3-4 min, 25%-95% B; 4-5.5 min, 95%-95% B; 5.5-6 min, 95%-5% B; 6-8 min, 5% B. The injection volume was set at 1 μL.

The mass spectrometer’s operating conditions were set as follows: a sheath gas flow rate of 35 Arb, an Aux gas flow rate of 10 Arb, a capillary temperature of 350 °C, and a quality scanning range from m/z 100 to 1500 Da. MRM mode, the collision energy were adjusted to 30, 40, and 50 eV. The spray voltage was set to 3.5 kV for positive ion mode and -2.8 kV for negative ion mode, respectively.

**S. 3 ICP-MS**

The metal elements within the BSFM were extracted in accordance with the previously described method (Zhou et al., 2023).

.

**TABLE S1** Determination of mineral element content (X ± S, *n* = 3)

| Element | Regression equation | r^2^ | μg/g |
| --- | --- | --- | --- |
| Mg | y=1000.6121x+1383.2336 | 0.9966 | 194.76±5.41 |
| Ca | y=580.7846x+799.4343 | 0.9971 | 1114.55±36.12 |
| Cr | y=32603.6331x+4670.2957 | 0.9971 | 7.74±0.049 |
| Fe | y=685.3681x+1820.3950 | 0.9970 | 212.77±4.28 |
| Cu | y=46778.9764x+6070.2060 | 0.9963 | 9.83±0.079 |
| Zn | y=5806.6062x+9629.2507 | 0.99512 | 31.22±0.21 |
| Se | y=47.1823x+2.0218 | 0.9988 | 2.13±0.052 |

Zhou, Y. M., Liu, J., Song, X. Q., Zhang, T., Li, H., Huang, L. Q., & Yuan Y. (2023). Melanin Prediction Model Study Based on‘Black Color’Digitization of Taihe Silky Chicken. *Chin Pharm J.* *58*(16): 1470-1477.

Sun, Y. Z., Xie, M. Y., Tu, Y. G., Tian, Y. G., Huang, L. H., Li, C.,& Wang, Y. X. (2008). Determination of Melanin from Black-bone silky fowl (*Gallus gallus domesticus* Brisson). *Journal of Instrumental Analysis*. *27*(12): 1363-1366.


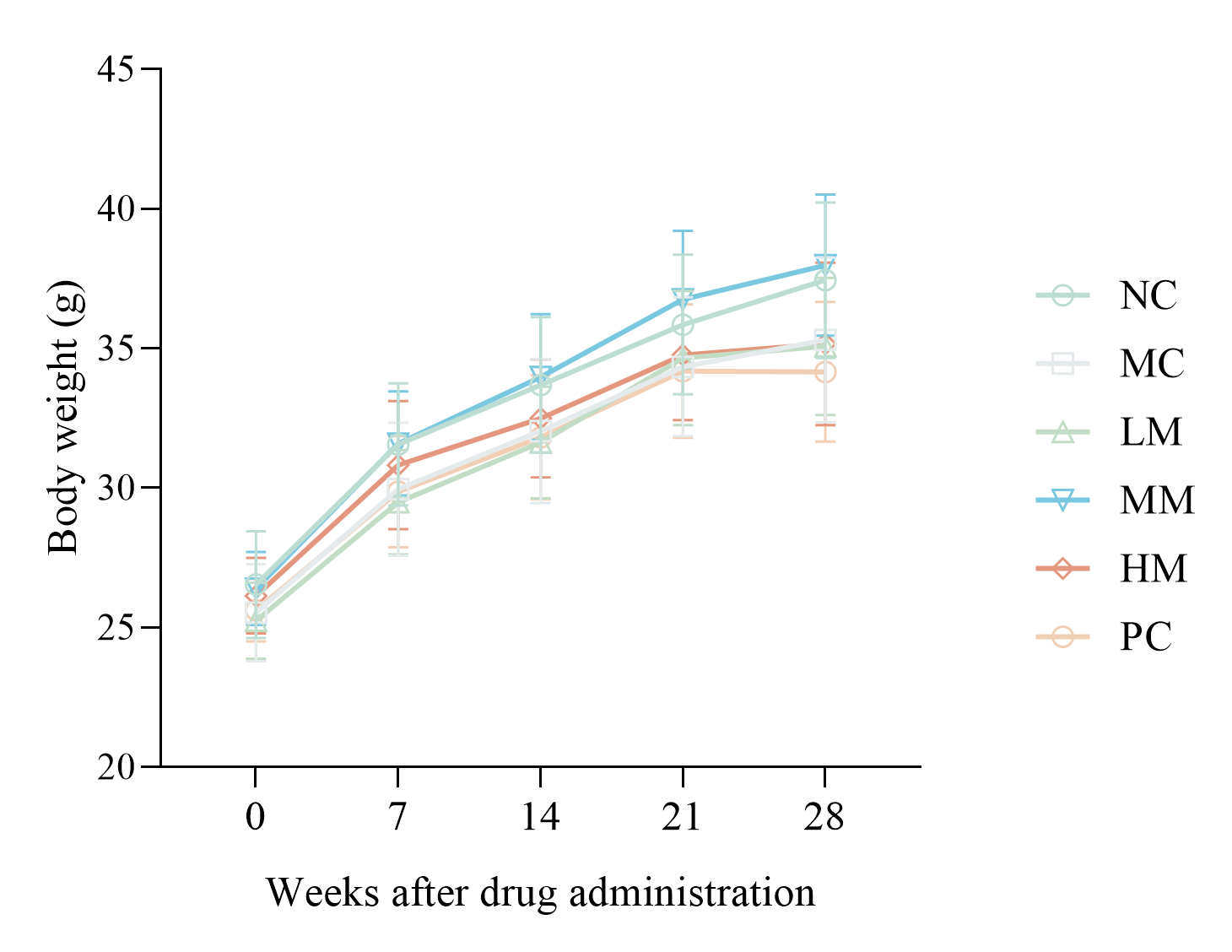


**FIGURE S1** Changes in body weight of mice.

**TABLE S2** Organ index

| **Group** | **Weight (g)** | **Liver index (mg/g)** | **Cardiac index (mg/g)** | **Spleen index (mg/g)** | **Kidney index (mg/g)** |
| --- | --- | --- | --- | --- | --- |
| NC | 37.44±2.94 | 49.85±3.86 | 6.18±1.36 | 4.18±0.79 | 14.90±1.59 |
| MC | 35.3±2.94 | 53.07±3.86 | 5.42±0.89 | 3.88±0.66 | 14.91±1.35 |
| PC | 34.16±2.50 | 54.98±3.98 | 5.26±0.37 | 9.90±2.94**** | 15.84±1.29 |
| LM | 35.07±2.45 | 50.07±4.50 | 5.16±0.89 | 3.97±0.87 | 14.51±1.58 |
| MM | 38.52±2.84 | 51.94±5.94 | 4.81±0.53 | 4.48±1.28 | 15.53±1.53 |
| HM | 35.16±2.90 | 49.62±2.72 | 5.08±0.93 | 4.66±1.26 | 14.12±1.58 |

Note: the value represents mean ± standard (*n* = 10). **p* < 0.05; *****p* < 0.001

**Uncropped gels for Western Blots in FIGURE 4**

**
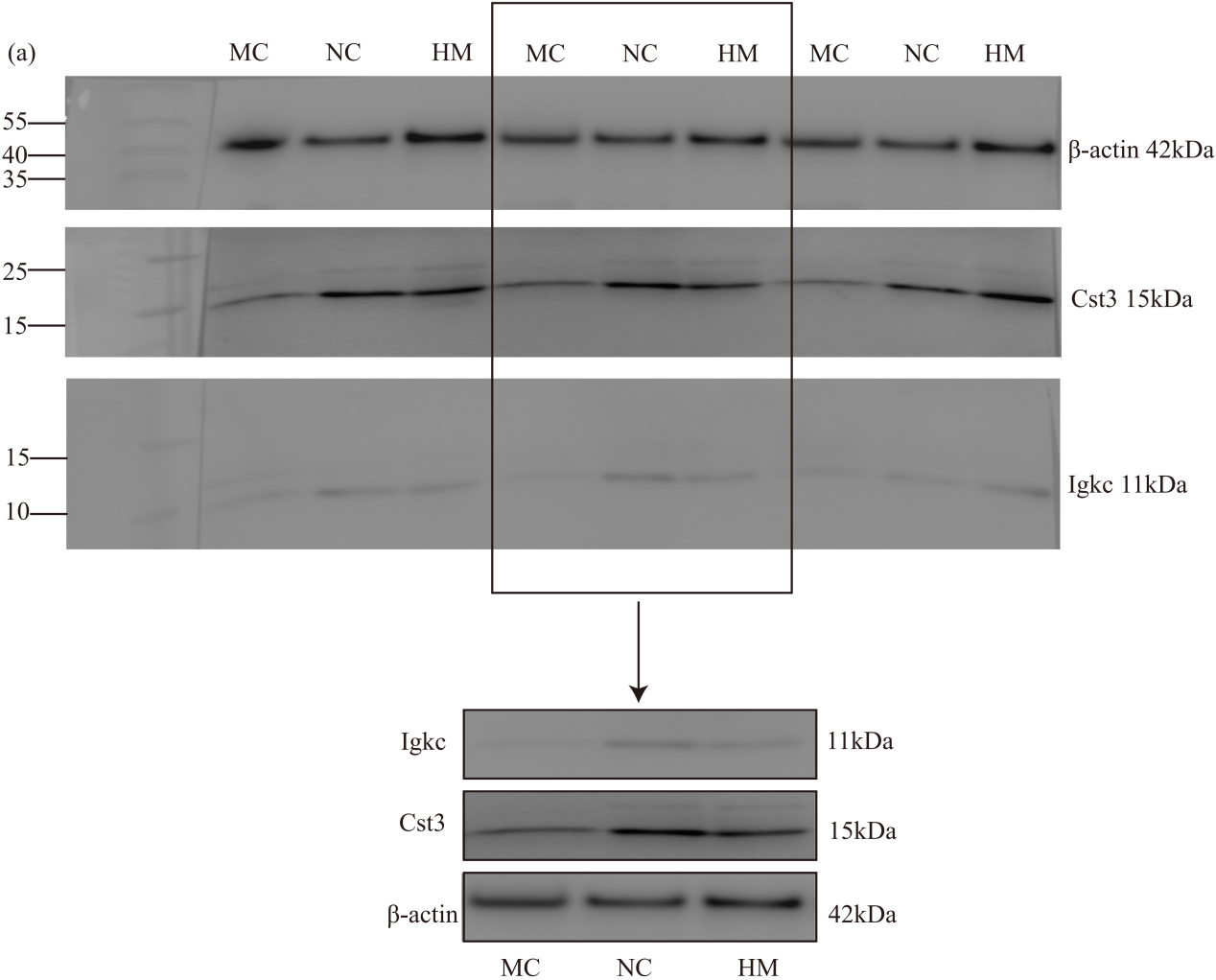
**

**TABLE S3** Expression of target proteins after application of BSFM treatment

| **Protein_ID** | **Name** | **Definition** | **NC-MC** | | | **MC-HM** | | |
| --- | --- | --- | --- | --- | --- | --- | --- | --- |
|  |  |  | **FC** | ***P*** | **Trend** | **FC** | ***P*** | **Trend** |
| ATP6_MOUSE | Mtatp6 | ATP synthase subunit a | 1.603 | 0.024 | Up | 0.654 | 0.014 | Down |
| A2APX3_MOUSE | Cst3 | Cystatin C (Fragment) | 1.678 | 0 | Up | 0.653 | 0.001 | Down |
| A0A0R4J1A7_MOUSE | Arid3a | AT rich interactive domain 3A (BRIGHT-like) | 1.809 | 0.05 | Up | 0.636 | 0.037 | Down |
| A0A0U1RNW0_MOUSE | Csnk1g1 | non-specific serine/threonine protein kinase | 1.866 | 0.017 | Up | 0.601 | 0.009 | Down |
| VTDB_MOUSE | Gc | Vitamin D-binding protein | 1.766 | 0.022 | Up | 0.601 | 0.029 | Down |
| Q3TE40_MOUSE | Rpa2 | Replication protein A2 | 1.722 | 0.006 | Up | 0.596 | 0.016 | Down |
| SPT6H_MOUSE | Supt6h | Transcription elongation factor SPT6 | 2.197 | 0.001 | Up | 0.59 | 0.017 | Down |
| SH3L3_MOUSE | Sh3bgrl3 | SH3 domain-binding glutamic acid-rich-like protein 3 | 1.784 | 0.001 | Up | 0.556 | 0.024 | Down |
| FLNC_MOUSE | Flnc | Filamin-C | 1.642 | 0.024 | Up | 0.553 | 0.011 | Down |
| A0A087WS98_MOUSE | Actr3 | ARP3 actin-related protein 3 (Fragment) | 1.513 | 0.003 | Up | 0.53 | 0.017 | Down |
| IGG2B_MOUSE | Ighg2b | Immunoglobulin heavy constant gamma 2B | 1.544 | 0.007 | Up | 0.51 | 0.024 | Down |
| VMA21_MOUSE | Vma21 | Vacuolar ATPase assembly integral membrane protein Vma21 | 1.83 | 0.014 | Up | 0.49 | 0.015 | Down |
| PGRP1_MOUSE | Pglyrp1 | Peptidoglycan recognition protein 1 | 1.937 | 0.001 | Up | 0.489 | 0.002 | Down |
| GPKOW_MOUSE | Gpkow | G-patch domain and KOW motifs-containing protein | 3.53 | 0.005 | Up | 0.476 | 0.028 | Down |
| CCL6_MOUSE | Ccl6 | C-C motif chemokine 6 | 3.372 | 0.007 | Up | 0.476 | 0.046 | Down |
| E9Q6E5_MOUSE | Srsf11 | Serine and arginine-rich splicing factor 11 | 1.801 | 0.004 | Up | 0.471 | 0.02 | Down |
| E9Q330_MOUSE | Pla2g7 | 1-alkyl-2-acetylglycerophosphocholine esterase (Fragment) | 1.693 | 0.02 | Up | 0.455 | 0.018 | Down |
| A0A5H1ZRK8_MOUSE | Igkc | Immunoglobulin kappa constant (Fragment) | 2.682 | 0.005 | Up | 0.45 | 0.001 | Down |
| A0A286YDL6_MOUSE | Camk2g | Calcium/calmodulin-dependent protein kinase II gamma (Fragment) | 2.635 | 0.002 | Up | 0.435 | 0.021 | Down |
| WDR55_MOUSE | Wdr55 | WD repeat-containing protein 55 | 1.886 | 0.047 | Up | 0.406 | 0.007 | Down |
| A0A0R4J1N8_MOUSE | Rdh13 | Retinol dehydrogenase 13 (all-trans and 9-cis) | 0.664 | 0.003 | Down | 1.616 | 0.033 | Up |
| E9Q2S9_MOUSE | Ccdc43 | Coiled-coil domain-containing protein 43 | 0.553 | 0.021 | Down | 2.317 | 0.002 | Up |
| MKLN1_MOUSE | Mkln1 | Muskelin | 0.635 | 0.027 | Down | 1.516 | 0.022 | Up |
| MITOK_MOUSE | Ccdc51 | Mitochondrial potassium channel | 0.517 | 0.04 | Down | 2.114 | 0.021 | Up |
| Q5SXC3_MOUSE | Vezf1 | Vascular endothelial zinc finger 1 (Fragment) | 0.463 | 0.02 | Down | 2.084 | 0.022 | Up |
| SFR1_MOUSE | Sfr1 | Swi5-dependent recombination DNA repair protein 1 homolog | 0.622 | 0.005 | Down | 1.862 | 0.005 | Up |
| RBM10_MOUSE | Rbm10 | RNA-binding protein 10 | 0.616 | 0.037 | Down | 2.093 | 0.016 | Up |
| TADA1_MOUSE | Tada1 | Transcriptional adapter 1 | 0.496 | 0 | Down | 1.559 | 0.008 | Up |
| RUSD4_MOUSE | Rpusd4 | Pseudouridylate synthase RPUSD4, mitochondrial | 0.567 | 0.011 | Down | 1.77 | 0.018 | Up |


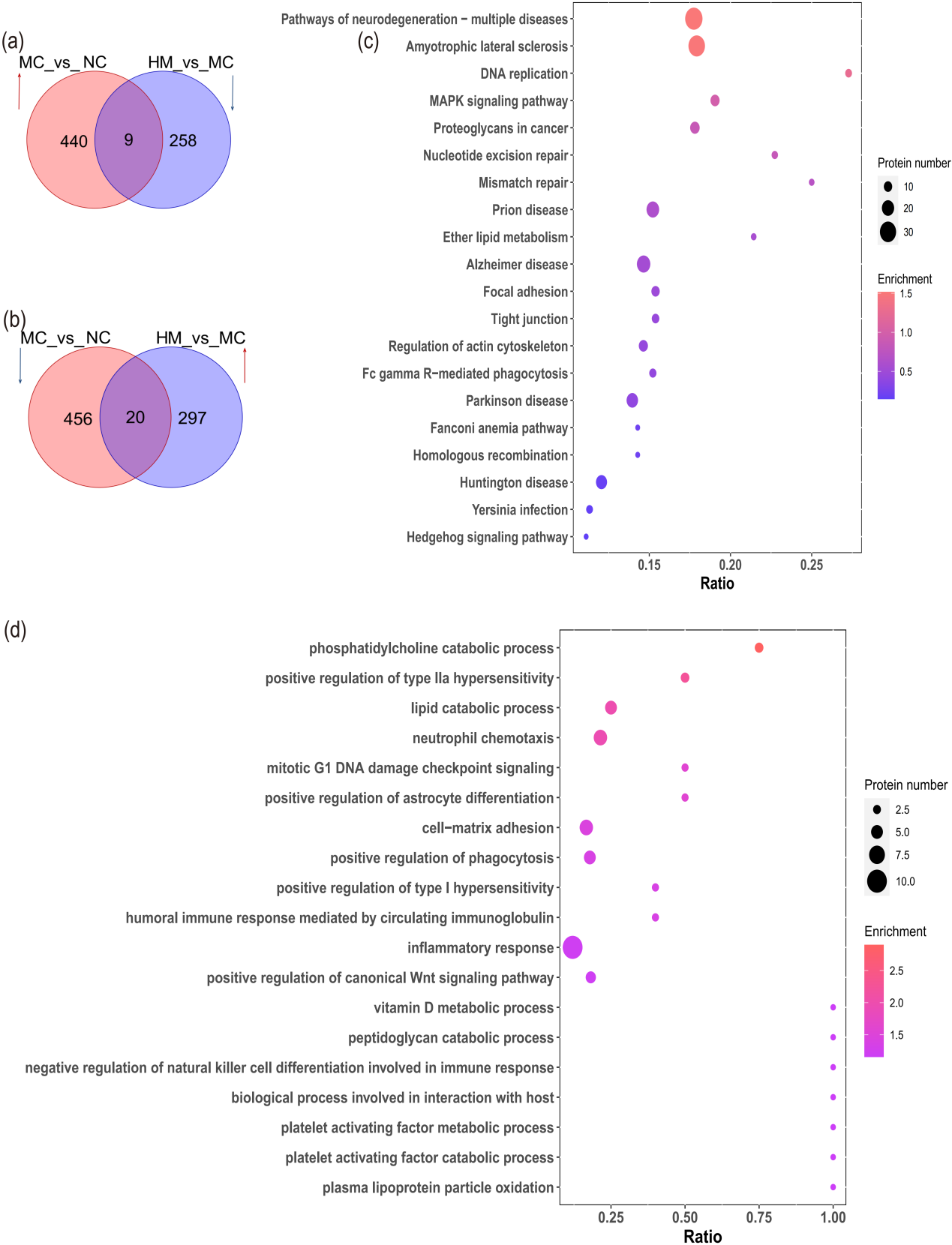


**FIGURE S2** Function analyses of common DEPs between NC vs MC and MC vs HM. (A) Overlaps between DEPs which were upregulated in MC group but downregulated in HM group. (B) Overlaps between DEPs which were downregulated in MC group but upregulated in HM group. (C) Biological process enrichment analysis of the common DEPs. (D) KEGG enrichment analysis of the common DEPs.

| **TABLE S4** Differential metabolites characterized in plasma and their change trends after melanin treatment | | | | | | | | | | | | | | |
| --- | --- | --- | --- | --- | --- | --- | --- | --- | --- | --- | --- | --- | --- | --- |
| Description | HMDB_ID | NC-MC | | | | | MC-HM | | | | NC-HM | | | |
|  |  | Ion Mode | VIP | FC | Trend | *P* | VIP | FC | Trend | *P* | VIP | FC | *P* | Trend |
|  |  |  |  |  |  |  |  |  |  |  |  |  |  |  |
| L-Threonine | HMDB0250801 | ESI- | 1.46 | 1.83 | Up* | 0.02 | 0.99 | 0.71 | - | 0.16 | 0.38 | 1.14 | - | 0.68 |
| 3-Hydroxycapric acid | HMDB0002203 | ESI- | 1.36 | 0.57 | Down** | 0 | 0.78 | 1.28 |  | 0.23 | 0.47 | 0.73 | - | 0.34 |
| 6-Phosphogluconic acid | HMDB0062800 | ESI- | 2.81 | 0.35 | Down* | 0.03 | 0.07 | 0.39 |  | 0.99 | 2.38 | 0.14 | - | 0.14 |
| Mono-ethylhexylphthalate | HMDB0013248 | ESI- | 2.46 | 3.62 | Up* | 0.02 | 0.47 | 0.95 |  | 0.6 | 1.82 | 3.43 | Up* | 0.02 |
| 1-[(2R,3S,5R)-3,4-Dihydroxy-5-(hydroxymethyl)oxolan-2-yl]pyrimidine-2,4-dione | HMDB0246131 | ESI- | 1.82 | 0.52 | Down** | 0 | 1.57 | 1.46 |  | 0.15 | 0.44 | 0.75 | - | 0.95 |
| Octanoylglucuronide | HMDB0010347 | ESI- | 2.24 | 2.65 | Up* | 0.02 | 2.52 | 0.4 | Down | 0.04 | 0.75 | 1.05 | - | 0.84 |
| Indole-3-aldehyde | HMDB0029737 | ESI- | 0.91 | 1.42 | - | 0.06 | 1.19 | 1.72 | Up* | 0.02 | 1.74 | 2.44 | Up** | 0 |
| 2-Methyladipic acid | HMDB0029167 | ESI- | 0.29 | 1.33 | - | 0.77 | 1.25 | 1.88 |  | 0.04 | 1.26 | 2.5 | - | 0.09 |
| GLUCURONATE | HMDB0000127 | ESI- | 1.2 | 0.6 | - | 0.08 | 1.23 | 1.75 |  | 0.05 | 0.07 | 1.06 | - | 0.93 |
| D-Gluconic acid sodium salt | HMDB0000625 | ESI- | 0.23 | 1.23 | - | 0.74 | 1.07 | 1.64 |  | 0.04 | 1.09 | 2.01 | - | 0.07 |
| D-Mannose-6-phosphate barium salt hydrate | HMDB0001078 | ESI- | 0.1 | 1.01 | - | 0.9 | 1.24 | 1.69 | Up** | 0 | 0.98 | 1.71 | - | 0.01 |
| Homovanillic acid sulfate | HMDB0011719 | ESI- | 0.28 | 0.86 | - | 0.7 | 1.42 | 2.11 | Up* | 0.04 | 0.97 | 1.8 | - | 0.06 |
| Octanoylglucuronide | HMDB0010347 | ESI- | 2.24 | 2.65 | Up* | 0.02 | 2.52 | 0.4 | Down* | 0.04 | 0.75 | 1.05 | - | 0.84 |
| Hesperetin | HMDB0030746 | ESI- | 0.9 | 1.32 | - | 0.23 | 0.59 | 1.23 | - | 0.57 | 1.2 | 1.63 | - | 0.1 |
| 2-AMINOISOBUTYRATE | HMDB0001906 | ESI+ | 1.59 | 0.46 | Down** | 0.01 | 1.57 | 2.05 |  | 0.05 | 0.49 | 0.94 | - | 0.7 |
| Gly-Gly | HMDB0011733 | ESI+ | 1.71 | 0.43 | Down** | 0.01 | 1.99 | 2.43 | Up* | 0.02 | 0.39 | 1.05 | - | 0.56 |
| N-Alpha-Acetyl-Ornithine | HMDB0003357 | ESI+ | 1.51 | 0.49 | Down** | 0.01 | 1.24 | 1.87 | Up** | 0.01 | 0.42 | 0.92 | - | 0.72 |
| Melatonin | HMDB0001389 | ESI+ | 0.35 | 1.85 | - | 0.99 | 3.28 | 4.41 |  | 0.01 | 1.25 | 2.7 | - | 0.17 |
| 2'-Deoxyinosine | HMDB0000071 | ESI+ | 1.05 | 2.91 | - | 0.42 | 0.66 | 1.23 | - | 0.27 | 0.06 | 0.99 | - | 0.82 |

**
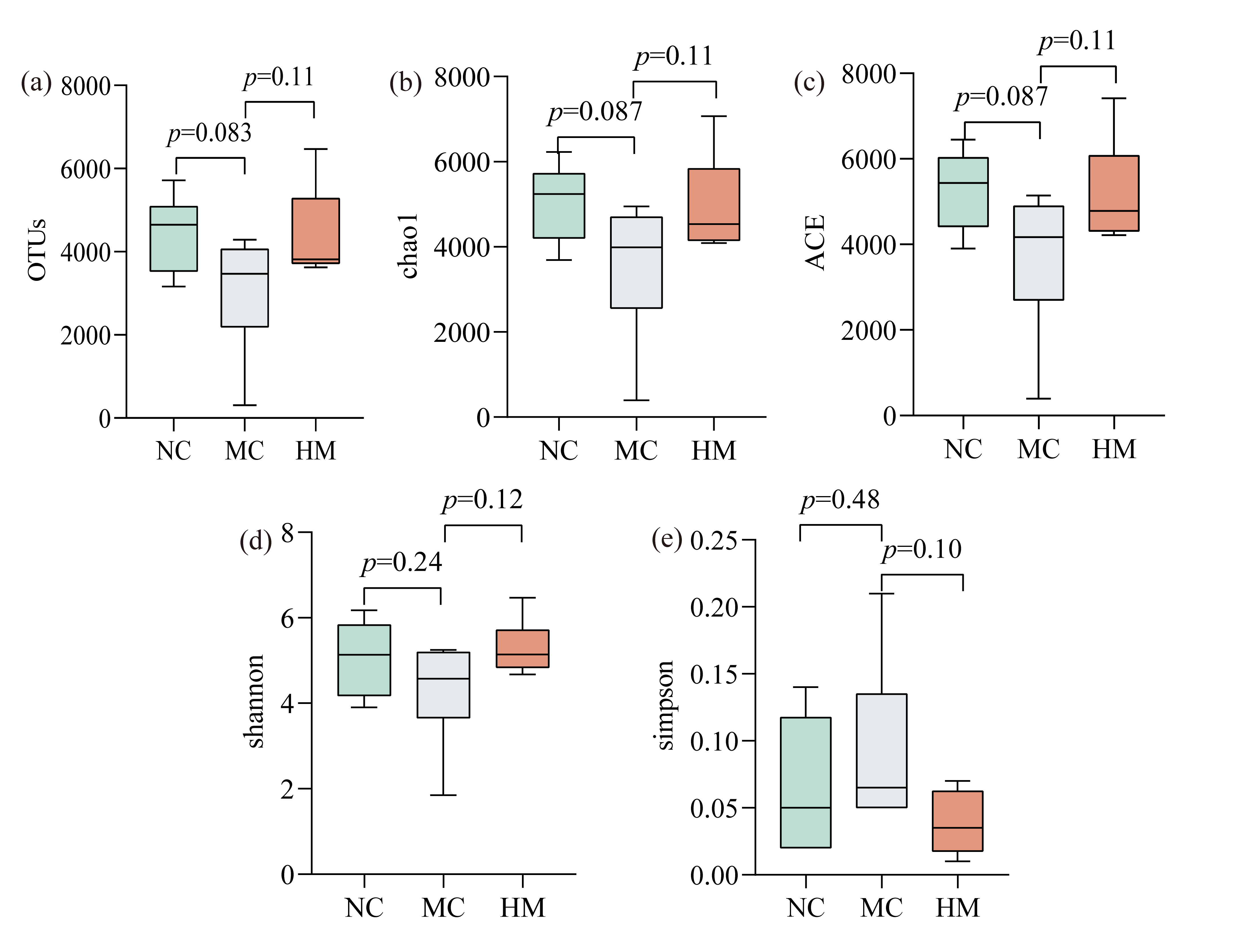
**

**FIGURE S3** Alpha diversity indices


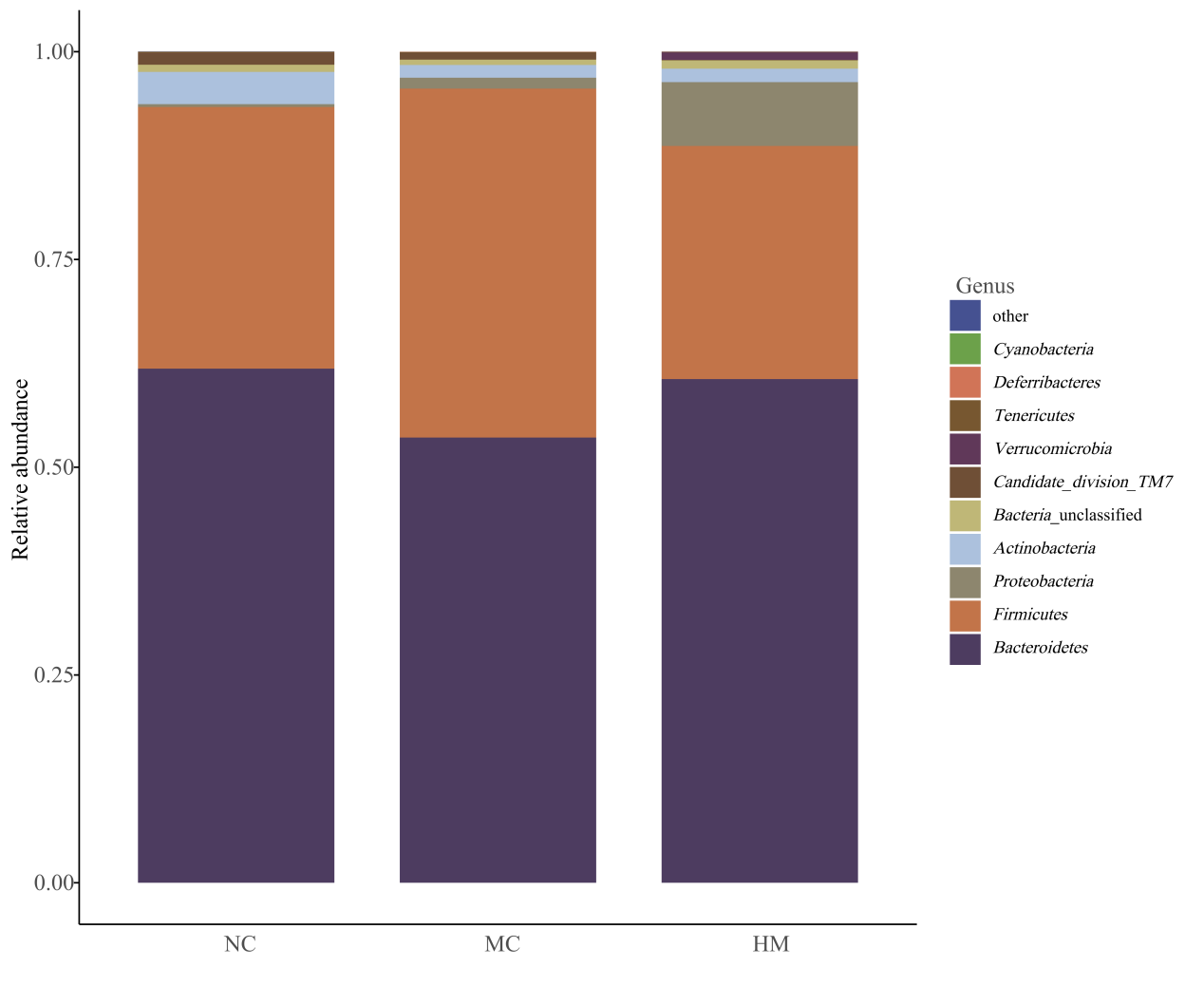


**FIGURE S4** phylum level

**TABLE S5** The abundance values of bacteria at the genus level

| **Group** | **Taxon** | | | | |  | |
| --- | --- | --- | --- | --- | --- | --- | --- |
|  | **S24-7** | **Olsenella** | **Prevotella** | **Parasutterella** | **hgcI** | **Lactococcus** | **Acetobacter** |
|  |  |  |  |  |  |  |  |
| NC1 | 0.282485 | 0.000066 | 0.000132 | 0.003396 | 0 | 0 | 0 |
| NC2 | 0.191705 | 0.000018 | 0.000212 | 0.000000 | 0 | 0 | 0 |
| NC3 | 0.199027 | 0.000029 | 0.000421 | 0.000412 | 0 | 0 | 0 |
| NC4 | 0.370261 | 0.000000 | 0.000568 | 0.000010 | 0 | 0 | 0 |
| NC5 | 0.336033 | 0.000034 | 0.000685 | 0.000011 | 0 | 0 | 0 |
| NC6 | 0.541865 | 0.000028 | 0.001104 | 0.000056 | 0 | 0 | 0 |
| MC1 | 0.119324 | 0 | 0.000092 | 0.000031 | 0 | 0 | 0 |
| MC2 | 0.245441 | 0 | 0.000105 | 0.000169 | 0 | 0 | 0 |
| MC3 | 0.098588 | 0 | 0.000029 | 0.000014 | 0 | 0 | 0 |
| MC4 | 0.372680 | 0 | 0.000075 | 0.000287 | 0 | 0 | 0 |
| MC5 | 0.004700 | 0.000124 | 0.000011 | 0.000011 | 0 | 0 | 0 |
| MC6 | 0.216416 | 0.000021 | 0.000174 | 0.000322 | 0 | 0 | 0 |
| HM1 | 0.320122 | 0.000615 | 0.000836 | 0.035977 | 0.000058 | 0.000673 | 0.000128 |
| HM2 | 0.358555 | 0.000196 | 0.000876 | 0.077477 | 0.000000 | 0.000546 | 0.000021 |
| HM3 | 0.287112 | 0.001026 | 0.000203 | 0.004955 | 0.000051 | 0.000368 | 0.000076 |
| HM4 | 0.392699 | 0.000110 | 0.002063 | 0.031457 | 0.000030 | 0.000431 | 0.000070 |
| HM5 | 0.313079 | 0.000196 | 0.005489 | 0.014030 | 0.000037 | 0.000478 | 0.000012 |
| HM6 | 0.246033 | 0.000153 | 0.000236 | 0.010430 | 0.000035 | 0.000460 | 0.000071 |

1. Correspondence

   Jianliang Li, National Resource Center for Chinese Materia Medica, China Academy of Chinese Medical Sciences, Beijing 100700, China. Email: [jianliang0320@126.com](mailto:jianliang0320@126.com)

   Yuan Yuan, Experimental Research Center, China Academy of Chinese Medical Sciences, Beijing 100700, China. Email: [y_yuan0732@163.com](mailto:y_yuan0732@163.com;) [↑](#footnote-ref-0)
